# Efficacy of a Food Supplement Containing *Lactobacillus acidophilus* LA14, Peptides, and a Multivitamin Complex in Improving Gastroesophageal Reflux Disease-Related Outcomes and Quality of Life of Subjects Showing Mild-to-Moderate Gastroesophageal Reflux Disease

**DOI:** 10.3390/nu16111759

**Published:** 2024-06-04

**Authors:** Francesco Tursi, Edoardo Benedetto, Amelia Spina, Ileana De Ponti, Fabio Amone, Vincenzo Nobile

**Affiliations:** 1 Research and Development, Complife Italia S.r.l., 27028 San Martino Siccomario, Italy; francesco.tursi@complifegroup.com (F.T.);; 2 GIGA-CP Italian Association for Primary Care Gastroenterology, 87036 Rende, Italy; 3 Nutratech S.r.l., Spin-Off of University of Calabria, 87036 Rende, Italy

**Keywords:** GERD, probiotics, bioactive peptides, QoL, clinical trial

## Abstract

Dietary interventions represent an interesting alternative to pharmacological treatments for improving the quality of life (QoL) of subjects suffering from gastroesophageal reflux disease (GERD). This randomized, double-blind, placebo-controlled study aimed to evaluate the efficacy of a food supplement (FS) containing a probiotic strain, bioactive peptides, and vitamins in relieving heartburn/dyspeptic symptoms in subjects with mild-to-moderate GERD. Fifty-six adult participants were randomly assigned to receive the placebo or the active FS for 28 days. Subjects were asked to record daily the frequency and intensity of heartburn episodes and the intake of over- the-counter (OTC) medications. GERD-QoL and self-assessment questionnaires were also completed every two weeks and at the end of the treatment, respectively. FS was effective in achieving a progressive and significant reduction of heartburn frequency and severity, with an intergroup significant difference at the end of the treatment period. FS group also reported a reduction in the OTC medication intake, whereas placebo administration did not modify the OTC intake. Results from the QoL and self-assessment questionnaires showed that FS administration achieved a progressive and statistically significant intragroup and intergroup improvement in the QoL score and a higher positive response with respect to the placebo treatment.

## 1. Introduction

The American College of Gastroenterology defines gastroesophageal reflux disease (GERD) as “chronic symptoms or mucosal damage incurred by the abnormal reflux of gastric contents into the oesophagus” that affects millions of people worldwide [1]. In fact, according to population-based studies, the pooled prevalence of GERD, defined by at least weekly GERD symptoms, was 13%, with a prevalence of more than 25% in some geographic regions [2,3], which means a significant economic burden in direct and indirect costs and adversely affects the quality of life of these subjects [4,5].

The pathophysiology of GERD is multifactorial [6] and it is linked to a misbalance between the aggressiveness of the gastric refluxate into the oesophagus or adjacent organs, and the failure of protective mechanisms, such as the buffering capacity of oesophageal epithelial cells [7], that results in symptoms such as heartburn, regurgitation, and chest pain. Symptomatic relief from GERD can be achieved by means of pharmacological treatments with proton pump inhibitors (PPIs), histamine-2 receptors antagonists, and antacids [8], each of which, being characterized by different mechanisms of action aiming at the neutralization of gastric acid and/or at the suppression of its production, must be specifically chosen according to the peculiar features of the disease. In fact, although PPIs represent the standard of care for GERD, they may not be effective for non-erosive reflux disease (NERD) [9]; PPIs and histamine-2 receptor antagonists show a limited efficacy when intermittently used [10] and exhibit relevant adverse effects; and antacids also present adverse effects and limitations of use in specific cases [11]. Therefore, an increasing demand for an alternative approach to the pharmacological treatment is currently driving research, and as diet has been theorized to be associated with the worsening of GERD symptoms, dietary interventions are one of the main treatment modalities recommended by international guidelines for GERD patients [12].

However, the efficacy of treatments based on avoiding trigger foods [1] is continuously analysed by systematic reviews and meta-analyses [13] that often point to limitations in these studies, mainly based on observational and epidemiological trials, and to the need of long-term Randomized Control Trials (RCTs) to confirm the effects of interventions. On the other hand, more comprehensive diet interventions, aimed at alleviating the severity and frequency of symptoms of GERD without or with limited adverse effects, represent a significant goal to pursue as they have a positive impact on the quality of life of subjects suffering from this disease [12].

In recent years, research has increasingly focused on identifying and characterizing the role of the human microbiota in the development of functional gastrointestinal disorders (FGIDs), with particular attention to GERD [14,15]. There is increasing evidence that both the onset and progression of GERD are influenced by the composition of the oesophageal microbiota and that commensal bacteria from the oropharynx, stomach, and colon play a role in modulating the pathogenesis of this disease [16]. These emerging hypotheses are based on observed changes in the composition of the oesophageal microbiota of subjects with GERD compared to healthy subjects [17]. In fact, taxonomic analyses have shown that in the healthy oesophagus, there is an abundance of Gram+ bacteria, while in the reflux oesophagus, Gram- bacteria predominate [18]. This imbalance towards a microbial population characterized by Gram- bacteria is associated with an increase in the antigenic potential of lipopolysaccharide (LPS), a bacterial component that is responsible for the inflammation of the oesophageal mucosa and the compression of the lower oesophageal sphincter, resulting in the relaxation of the latter and in the consequent increase in gastric acid contents in the oesophagus [16].

This evidence has stimulated scientific interest about the possible efficacy of probiotic-based supplement approaches to improve GERD symptoms by restoring the balance and composition of the oesophageal, gastric, and intestinal microbiota [19].

The administration of probiotic bacteria, especially belonging to the Lactobacillus genus, was found to be effective in accelerating and improving gastric digestive processes through an increment in the production of pepsinogen, which was found to be directly related to GERD symptoms [20]. Furthermore, it has been suggested that probiotics could improve disease symptoms by modulating barrier function and immune response [21].

Probiotic supplementations also have a positive effect on the composition of the microbial ecosystem by improving the Gram+/Gram− ratio, by reducing the percentage of *Prevotella* spp., which are the main colonizers of the gastric and oesophageal flora in patients with functional dyspepsia [22].

The growing scientific evidence supports the efficacy of bioactive peptides derived from the bacterial fermentation of soy as alternative treatments for GERD; such bioactive peptides are already recognized as compounds with beneficial physiological properties that include lipid lowering (hypocholesterolemic, hypotriglyceridaemic, and anti-obesity), anti-diabetic, antitumour, hypotensive, anti-inflammatory, and antioxidant effects in various experimental models [23]. Clinical evidence has shown that supplementation with fermented soy peptides can alleviate acid reflux [24] and reduce heartburn in subjects with mild-to-moderate heartburn, improving their quality of life [25].

This preliminary interventional study was designed to assess the effectiveness of a treatment involving a dietary supplement containing a specific probiotic strain, *Lactobacillus acidophilus* LA14, and bioactive peptides, derived from the fermentation of soy proteins by *Lactobacillus bulgaricus*, in improving GERD-related outcomes and quality of life (QoL) in subjects showing mild-to-moderate GERD.

## 2. Materials and Methods

### 2.1. Study Participants

A total of 56 healthy male and female subjects (18–50 years old) were enrolled in the study by the gastroenterologist if they experienced mild or moderate heartburn according to The Short-Form Leeds Dyspepsia Questionnaire [26], and used over-the-counter (OTC) medications for heartburn, food supplements, or dietary manipulation to relieve heartburn symptoms during the previous 3 months. All subjects expressed their willingness not to use other products apart from the test product throughout the study period and antacids (to be reported), not to vary the normal daily routine (i.e., lifestyle, physical activity, usual diet, fluid intake, etc.), to keep a stable pharmacological therapy (except for the pharmacological therapy reported in the exclusion criteria) for at least one month without any changes expected or planned during the study, including effective contraception (oral/not oral) therapy. Exclusion criteria were the following: alimentary/eating disorders (i.e., bulimia, psychogenic eating disorders, etc.); severe heartburn problem during the week prior to the study; previous or current treatment for any gastrointestinal diseases or illnesses; a diagnosis of Barrett’s oesophagus; previous upper gastrointestinal surgery; clinically significant gastrointestinal bleeding within the previous three months; oesophagitis not related to acid reflux; known hypersensitivity or allergy to any of the active ingredients; known food intolerance or food allergy; bleeding disorder; Zollinger–Ellison syndrome; duodenal/gastric ulcer and upper gastrointestinal malignancy; pharmacological treatments (abuse of FANS, antibiotics, etc.) known to interfere with the tested product; unstable medical diseases (cardiac arrhythmias or ischemia, uncontrolled hypertension and hypotension, diabetes mellitus, and kidney failure); and pregnancy or breastfeeding.

All subjects were informed about the nature, purpose, benefits, and risks of participation in the study and were asked to read and sign the informed consent form (ICF).

### 2.2. Study Design 

A 4-week, randomized, double-blind, placebo-controlled, parallel-group study was conducted in Nutratech Srl, Rende (CS), Italy, from July to December 2023. The study was approved by the Independent Ethical Committee for Non-Pharmacological Clinical Study Trials, Genova, Italy (Ref. 23/10).

Subjects were recruited from the company database and attended the medical centre on day −7 for signing the ICF and for verifying eligibility to the study through the Short-Form Leeds Dyspepsia Questionnaire, that included subjects who, over 2 months, experienced heartburn frequency and an interference of heartburn with their normal activities, with a total score 1–6 out of 8. Demographic data and medical history were collected, and all subjects were provided with a 7-day food diary and a 7-day symptom questionnaire. Eligibility was confirmed a week later, on day 0, by interviewing subjects with the GERD-QoL [27,28] and enrolling subjects with a score >8 and <15. Subjects were randomized to receive the food supplement (FS) or the placebo PL), and received the amount of assigned product for 14 days of use, a 14-day daily diary for compliance/tolerance/dietary habits, and a 14-day symptom questionnaire. Treatment tolerance and compliance to study protocol and to product intake were checked on day 14; then, all subjects were interviewed again with the GERD-QoL, filled in the self-assessment questionnaire, and received the amount of assigned product for a further 14 days of use, a 14-day daily diary for compliance/ tolerance/dietary habits, and a 14-day symptom questionnaire. At the final visit, on day 28, diaries were collected and treatment tolerance and compliance to study protocol and to product accountability were checked; finally, all subjects were interviewed with the GERD-QoL and filled in the self-assessment questionnaire.

### 2.3. Primary and Secondary Outcomes of the Study

The primary objective of the study was to evaluate the relief of heartburn symptoms and other dyspeptic symptoms in subjects with mild-to-moderate GERD; the secondary objective of the study was to evaluate their heartburn-related quality of life.

#### 2.3.1. Quality of Life Questionnaire (GERD-QoL)

The GERD-QOL [27,28] is a short and validated self-assessment questionnaire that assesses the presence of GERD. It is a 6-item questionnaire (scale: from 0 to 3 for the four positive predictors of GERD—0 days has a score of ‘0’, 1 day has ‘1’, 2–3 days has ‘2’, 4–7 days has ‘3’, or in reversed order for the two negative predictors of GERD) and has been developed with questions from the Reflux Disease Questionnaire (RDQ), the Gastrointestinal Symptom Rating Scale (GSRS), and the Gastrointestinal Symptom Scale (GIS), which are all validated disease-specific questionnaires. The first two questions (1 and 2) are positive predictors of GERD, and a higher score suggests higher symptom frequency. Questions 3 and 4 address dyspeptic symptoms that lower the probability of GERD; they are negative predictors of GERD. The last two questions (5 and 6) assess the impact of GERD symptoms on people’s life habits and are also positive predictors of GERD.

Frequency (n° of episodes) and heartburn severity were scored daily using a Likert-like scale (1 = no symptoms to 5 = severe discomfort); the usage of OTC antiacid drugs was recorded in the diary starting from T-7 until T28.

#### 2.3.2. Self-Assessment Questionnaire

On day 14 and day 28, subjects were asked to score their perceived efficacy of treatments and other product properties for comparison to the initial score (10 questions).

### 2.4. Products

The food supplement (Pilorex^®^; Bromatech, Milan, Italy) composition was as follows: non-GMO soy proteins fermented by *Lactobacillus Bulgaricus* (77%); Sodium carboxymethylcellulose, Microcrystalline cellulose, magnesium salts of fatty acids, and Silicon dioxide as anti-caking agents; Calcium carbonate; *Lactobacillus acidophilus* LA14; Vitamin C; Niacin (Vitamin PP); Calcium pantothenate; Coating agent: hydroxypropyl cellulose; Vitamin B2 (riboflavin); Vitamin B6 (pyridoxine hydrochloride); Vitamin B1 (thiamine mononitrate); and Folic acid.

Placebo tablets contained Microcrystalline cellulose (cellulose gel), calcium phosphates, fatty acids, iron oxides, and Hydroxypropyl cellulose.

Subjects were instructed to intake four tablets per day, two in the morning upon waking up before breakfast, and two after dinner/before going to bed, with a glass of water.

All subjects were provided with anonymous packs containing FS or PL, randomly assigned using a restricted randomization list created by a biostatistician using PASS 2008 (PASS, LLC. Kaysville, UT, USA) statistical software. The randomization sequence was stratified using “Efron’s biased coin” algorithm (1:1 allocation ratio). Neither the subjects nor the personnel involved in the study were aware of the FS/PL distribution list.

### 2.5. Statistical Analysis

Results, graphs, and statistical analyses refer to per-protocol population (PP). An appropriate statistical model was applied based on data distribution. The intragroup statistical analysis was carried out using Friedman’s test on variations vs. T0. The intergroup statistical analysis was carried out using Kruskal–Wallis test on variations vs. T0. The statistical software used was NCSS 10 (version 10.0.7 for Windows; NCSS, Kaysville, UT, USA).

## 3. Results

**Figure 1:**
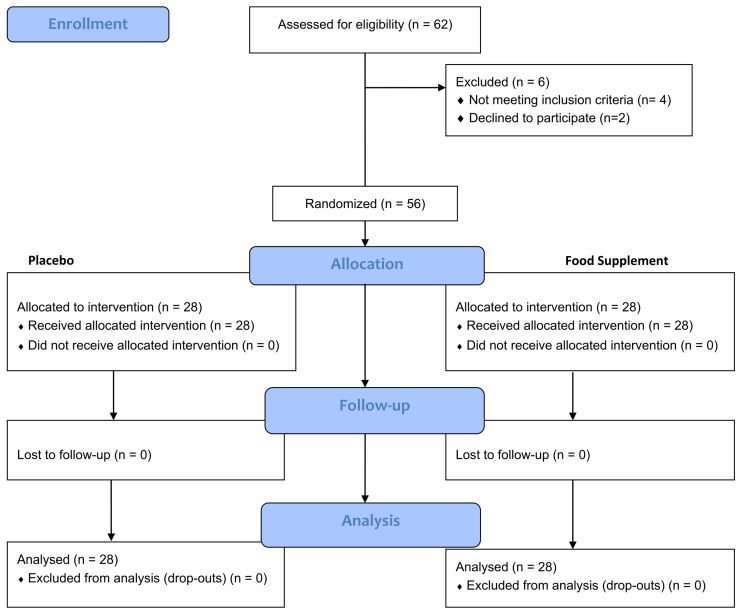
Participant flow diagram.

Treatments were found to be well tolerated by all subjects, and no adverse event either related or not related to the tested products occurred during the study period. No drop-out was recorded. All subjects were compliant with the treatment scheme, and no major or minor protocol deviation was recorded in the treatment regimen for both study groups. Efficacy analysis was based on the per-protocol population on the whole panel of 28 subjects per group (Figure 1).

### 3.1. Heartburn Frequency and Severity

No statistically significant intergroup difference was recorded in the initial heartburn frequency and the initial heartburn severity (Table 1), supporting the homogeneous composition of the two groups. PL administration did not result in any relevant difference in either parameter throughout the study; instead, the FS treatment resulted in a progressive and statistically significant (*p* < 0.001) intragroup reduction in heartburn frequency, that at T14 and T28, accounted, respectively, for −23.2% and −27.4% of the initial values, and in a statistically significant intergroup difference at T14 (*p* < 0.05) and at T28 (*p* < 0.001).

A similar trend was achieved with regard to heartburn severity: FS administration achieved a progressive and statistically significant intragroup reduction in severity at T14 (*p* < 0.01) and T28 (*p* < 0.001), that accounted, respectively, for −15.4% and −27.1% of the initial values, and in a statistically significant intergroup difference at T28 (*p* < 0.001).

**Figure 2:**
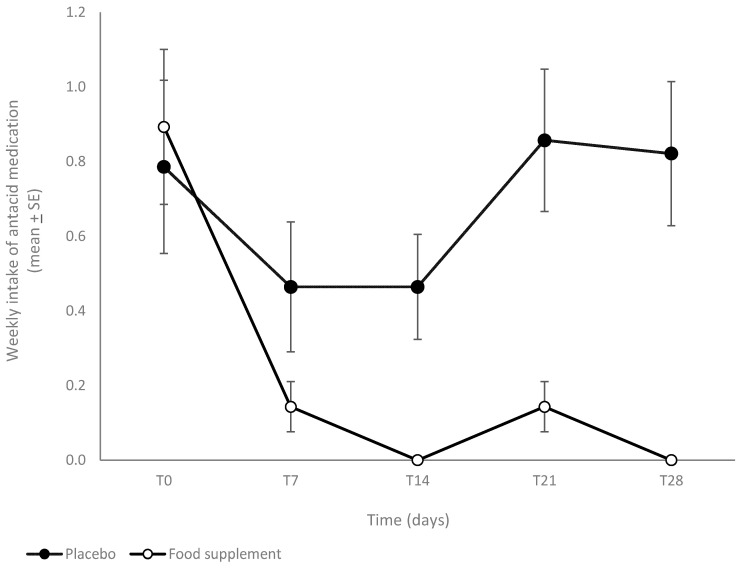
Weekly intake of antacid medication. Data are reported as mean ± SE.

The FS group reported a reduction in the weekly intake of OTC antacids with respect to the placebo group (Figure 2); in fact, starting from a similar weekly intake of OTC antacid (0.8 ± 0.23 and 0.9 ± 0.20, respectively, for the PL and FS groups), an almost complete reduction in the drug uptake was achieved in the FS group already after 7 days of treatment, and it was maintained throughout the fermented soy intake, whereas the PL group showed an initial reduction (at T7 and T14) and a rebound on the number of medications per week, suggesting an initial placebo effect in those subjects.

### 3.2. GERD-QoL Questionnaire

**Figure 3:**
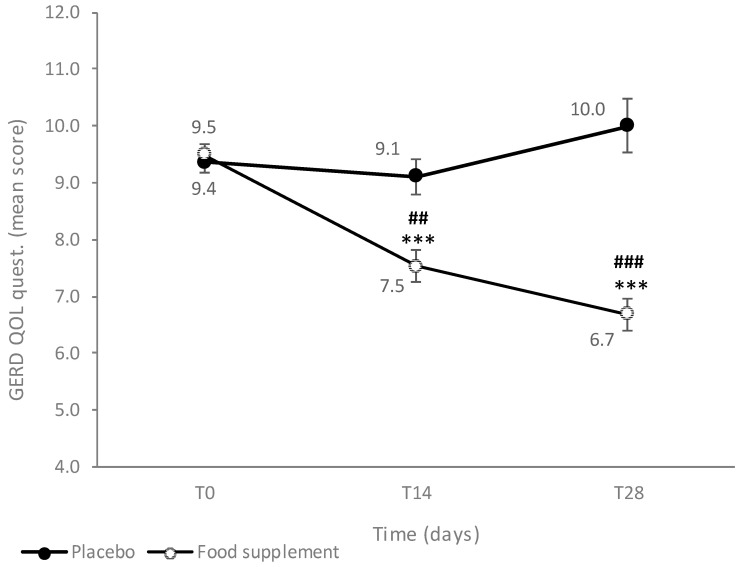
GERD-QoL questionnaire: scores are reported as mean value ± SE. Intragroup statistical analysis using Friedman’s test and intergroup statistical analysis # using the Kruskal–Wallis test are reported as follows: ## *p* < 0.01; ***### *p* < 0.001.

The initial mean score of GERD-QoL did not differ between the two groups: 9.4 ± 0.2 and 9.5 ± 0.2, respectively, for the PL and FS groups (Figure 3). The administration of the FS achieved a progressive and statistically significant (*p* < 0.001) intragroup reduction in the score, that, respectively at T14 and T28, accounted for −20.1% and −29.1% of the initial values, and in a statistically significant intergroup difference at T14 (*p* < 0.01) and at T28 (*p* < 0.001). A non-definite trend was recorded in subjects receiving the PL treatment, as after an initial slight reduction in the score, a rebound was recorded at T28.

### 3.3. Self-Assessment Questionnaire

The positive perception of the treatment’s efficacy expressed after 14 days was found to be lower in subjects receiving the placebo with respect to subjects receiving the soy ferment treatment; the mean percentage of positive responses (completely agree + agree) to the 10 questions submitted was found to be 57.68 ± 3.68 for the placebo and 82.15 ± 2.62 for the soy ferment group. The difference increased at T28, as positive responses with respect to the beginning of the treatment decreased in the PL group, whereas higher positive responses were expressed by the subjects receiving the FS treatment: 47.14 ± 3.19 for the placebo and 90.31 ± 1.20 for the soy ferment group.

## 4. Discussion

The management of the symptoms of GERD encompass mainly pharmaceutical interventions, none of which resolutive due to different pathophysiological features of the disease, and also due to adverse or side effects and limitations of using such products; surgical treatments [29], dietary and lifestyle interventions [30] are also applied, and the last two approaches are receiving increasing attention by patients, who always look for a safer treatment that concurrently allows an *ad personam* modulation.

Results achieved in the present RCT study showed that the administration of a food supplement containing non-GMO soy proteins fermented by *Lactobacillus bulgaricus*, a micro-encapsuled *Lactobacillus acidophilus LA14*, Vitamin C, Niacin, Calcium pantothenate, Vitamin B1-B2-B6, and Folic acid, was effective in achieving a significant reduction in the typical GERD symptoms such as heartburn severity and heartburn frequency in a population of adults with a mild-to-moderate GERD, a result that correlates with the improvement in the quality of life, and in the reduction in the weekly intake of OTC drugs recorded by these subjects.

Probiotics and fermented soy proteins have already been evaluated in vivo for their ability to improve upper and lower GI tract condition [31,32]. Indeed, there is highly supportive evidence that probiotic improve overall GI symptoms [19,33] and abdominal pain in irritable bowel syndrome (IBS) [34], and reduce the risk of antibiotic-associated diarrhoea [35] and side effects associated with *Helicobacter pylori* eradication therapy [36,37]. On the other hand, the fermented soy protein exerts protective effects on gastric injury [38], may lessen acid reflux [24], and reduce heartburn occurrence in individuals who experienced mild or moderate heartburn [25].

Results from this study provide further support to the efficacy of using probiotic-based supplements in improving GERD symptoms through the re-establishment of the balance and composition of the oesophageal, gastric, and intestinal microbiome [39]. Indeed, the results could be related to the combined effect of *Lactobacillus strain* [20,21,22] and bioactive peptides obtained from soy proteins fermentation [24,25] used, through the amelioration of the Gram+/Gram− ratio, the mucosal function, and the immune response [21], and pepsinogen secretion [22,23].

The present RCT has some limitations, such as the small number of subjects involved, although such limitation was related to the preliminary investigation design; the duration of the treatment, that did not allow the researchers to detect significant intergroup differences, although a progressive improvement in the frequency and severity of GERD symptoms was achieved and subjects appreciated the reduction in OTC intake; and the prevalence of subjects presenting mild symptoms of GERD.

## 5. Conclusions

In conclusion, the administration of probiotics and fermented soy proteins could be useful in ameliorating the biodiversity and composition of the oesophageal, intestinal, and gastric microbiota, and, consequently, the GI tract homeostasis in subjects with GERD. Nevertheless, further clinical assays are required to investigate the actual effectiveness and mechanisms of action of strain-specific probiotics and fermented soy proteins in the treatment of GERD, especially in a larger number of subjects.

## Figures and Tables

**Table 1:** Heartburn frequency and heartburn severity. Data are reported as mean value ± SE. Intragroup statistical analysis * using the Friedman test and intergroup statistical analysis # using the Kruskal–Wallis test are reported as follows: # *p* < 0.05; ** *p* < 0.01; ***### *p* < 0.001.

	**Heartburn Frequency**			
	**Placebo**		**Food Supplement**	
**Time**	**Score**	**Tx − T0**	**Score**	**Tx − T0**
T0	1.90 ± 0.1		1.77 ± 0.1	
T14	1.78 ± 0.1	−0.12 ± 0.08	1.30 ± 0.1 ***	−0.47 ± 0.09 ^#^
T28	1.88 ± 0.1	−0.01 ± 0.09	1.24 ± 0.1 ***	−0.53 ± 0.08 ^###^
	**Heartburn Severity**			
	**Placebo**		**Food Supplement**	
**Time**	**Score**	**Tx − T0**	**Score**	**Tx − T0**
T0	2.17 ± 0.1		2.20 ± 0.1	
T14	2.06 ± 0.1	−0.10 ± 0.08	1.83 ± 0.1 **	−3.7 ± 0.11
T28	2.16 ± 0.1	−0.01 ± 0.09	1.60 ± 0.1 ***	−6.1 ± 0.10 ^###^

## Data Availability

The datasets presented in this article are not readily available because they contain know-how and are the property of the sponsor of the study (Bromatech, Milan, Italy). Requests to access the datasets should be directed to the corresponding author.
